# Effect of Adding Midazolam to Dual Prophylaxis for Preventing Postoperative Nausea and Vomiting

**DOI:** 10.3390/jcm10214857

**Published:** 2021-10-22

**Authors:** Jiyoung Lee, In Kyong Yi, Jung Youn Han, Eun Duc Na, Chunghyun Park, Jong Yeop Kim

**Affiliations:** 1 Department of Anesthesiology and Pain Medicine, CHA Bundang Medical Center, CHA University, 59 Yatap-ro, Bundang-gu, Seongnam 13496, Korea; jlee0616@cha.ac.kr (J.L.); hanjungyoun90@gmail.com (J.Y.H.); anesthpark@chamc.co.kr (C.P.); 2 Department of Anesthesiology and Pain Medicine, Ajou University School of Medicine, 164 World Cup-ro, Yeongtong-gu, Suwon 16499, Korea; lyrin01@aumc.ac.kr; 3 Department of Obstetrics and Gynecology, CHA Bundang Medical Center, CHA University, 59 Yatap-ro, Bundang-gu, Seongnam 13496, Korea; ned@chamc.co.kr

**Keywords:** antiemetics, midazolam, postoperative nausea and vomiting, prophylaxis, gynecology, laparoscopy

## Abstract

Multimodal prophylaxis for postoperative nausea and vomiting (PONV) has been recommended, even in low-risk patients. Midazolam is known to have antiemetic properties. We researched the effects of adding midazolam to the dual prophylaxis of ondansetron and dexamethasone on PONV after gynecologic laparoscopy. In this prospective, randomized, double-blinded trial, 144 patients undergoing gynecological laparoscopic surgery under sevoflurane anesthesia were randomized to receive either normal saline (control group, *n* = 72) or midazolam 0.05 mg/kg (midazolam group, *n* = 72) intravenously at pre-induction. All patients were administered dexamethasone 4 mg at induction and ondansetron 4 mg at the completion of the laparoscopy, intravenously. The primary outcome was the incidence of complete response, which implied the absence of PONV without rescue antiemetic requirement until 24 h post-surgery. The complete response during the 24 h following laparoscopy was similar between the two groups: 41 patients (59%) in the control group and 48 patients (72%) in the midazolam group (*p* = 0.11). The incidence of nausea, severe nausea, retching/vomiting, and administration of rescue antiemetic was comparable between the two groups. The addition of 0.05 mg/kg midazolam at pre-induction to the dual prophylaxis had no additive preventive effect on PONV after gynecologic laparoscopy.

## 1. Introduction

Postoperative nausea and vomiting (PONV) are common and severe complications following an operation and are often associated with delayed recovery and extended length of hospital stay [1]. Gynecologic laparoscopy, in particular, is correlated with a relatively higher incidence of PONV (approximately 80%) if no prophylactic antiemetics are administered [2]. The recognized risk factors for PONV are female sex, use of postoperative opioids including patient-controlled analgesia (PCA), volatile anesthesia, gynecologic surgery, and increased intra-abdominal pressure during laparoscopy [3]. Since the etiology of PONV is multifactorial and the mechanism is complex, multimodal prophylaxis has been recommended in high-risk patients [4]. The key concept of multimodal prophylaxis is that a combination of antiemetics of different classes acts on different receptors. Habib and Gan [5] revealed that in patients who did not respond to prevention using a certain class of antiemetics, administering an antiemetic of another class was more effective as a rescue agent than the agent used initially for prophylaxis. Many previous studies have reported that multimodal prophylaxis was superior to single-agent prophylaxis [6,7]. According to a new PONV guideline published in 2020, the indication for multimodal prophylaxis has been expanded, and it is now recommended to combine two or more agents even for low-risk patients [4]. The most commonly used regimen of multimodal prophylaxis consists of a 5-hydroxytryptamine (5-HT3) receptor antagonist and dexamethasone [8].

Midazolam, a short-acting benzodiazepine, is known to have antiemetic properties [9]. According to previous studies, perioperative administration of intravenous (IV) midazolam is associated with a decrease in PONV incidence [10,11]. Midazolam combined with a single-agent antiemetic therapy was more effective than the single-agent therapy alone [10,11]. Combining ondansetron and midazolam is superior to ondansetron alone [12], and combining dexamethasone and midazolam is superior to dexamethasone alone [13]. The mechanism of action of midazolam is presumed to be distinct from that of the 5-HT3 receptor antagonists and dexamethasone. However, it is not known whether midazolam has an additional preventive effect on PONV when used in conjunction with the current multimodal prophylaxis, which already comprises two or more agents.

In this study, our main hypothesis was that adding midazolam to dual prophylaxis would be more efficient in preventing PONV than dual prophylaxis alone. We researched using a randomized, double-blind, placebo-controlled trial to elucidate whether adding midazolam to dual prophylaxis of dexamethasone and ondansetron would be more efficacious than dual prophylaxis alone for preventing PONV after gynecologic laparoscopy.

## 2. Materials and Methods

This trial was performed with the approval of the institutional review board of the CHA Bundang Medical Center, CHA University, Seongnam, Korea (Approval No. CHAMC 2018-04-025, Approval Date 24 May 2018) and following the Helsinki Declaration, from 15 October 2018, to 24 September 2019. Before the enrollment of the first patient, we registered this study at the Clinical Research Information Service (https://cris.nih.go.kr, Registration No. KCT0002930, Registered Date 17 June 2018). Signed informed consent was acquired from every eligible participant. Patients 19–65 years of age with an American Society of Anesthesiologists (ASA) physical status classification of <III, who were planned for an elective gynecologic laparoscopy, were included. Patients with a history of anticancer chemotherapy, alcohol or drug abuse, chronic opioid use, antiemetic intake 24 h before laparoscopy, allergy to the study drugs, hepatic (liver enzymes > double the normal value) or renal (serum creatinine > 1.6 mg/dL) insufficiency, change to laparotomy, child-bearing or breastfeeding, borderline QTc prolongation (>450 ms), diabetes mellitus, infectious diseases, gastritis or gastric ulcer, or body mass index > 35 kg/m^2^, and those who could not score PONV or pain were excluded. Simple randomization in a one-to-one ratio was created by a computer-developed random number table. The eligible subjects were designated to either the control group or the midazolam group based on the randomization with closed and opaque envelopes.

Patients entered the operating room without premedication and conventional monitoring methods including pulse oximetry, electrocardiography, and noninvasive blood pressure monitoring were applied. Following three minutes of preoxygenation, IV midazolam 0.05 mg/kg was injected into the subjects in the midazolam group, and an identical volume of normal saline was given to those in the control group. The syringes carrying midazolam or normal saline were indistinguishable and had no medicine identification. The injection was administered by a registered nurse who was not associated with the medication preparation. Subsequently, we induced anesthesia using propofol 2.0 mg/kg, fentanyl 1.0 μg/kg, and rocuronium 0.6–0.8 mg/kg for tracheal intubation. Mechanical ventilation with 40% oxygen in the air was used to sustain an end-tidal partial pressure of CO_2_ between 35 and 40 mmHg. Sevoflurane 2–3 vol% was used to maintain the anesthesia. If the pulse rate or blood pressure of the patient had increased to >20% of the baseline value despite increasing sevoflurane of 3 vol%, fentanyl 50 μg would be administered, additionally. An anesthesiologist who was unaware of the randomization performed the anesthesia induction, maintenance, and emergence. For multimodal prophylaxis of PONV, IV dexamethasone 4 mg was administered shortly after the anesthetic induction. At the completion of each surgery, IV ondansetron 4 mg was injected. Every subject was also administered IV-PCA (Accufuser; Wooyoung Meditech, Seoul, Korea) containing sufentanil 2.5 μg/kg, ketorolac 90 mg, ondansetron 12 mg, and normal saline in a volume of 100 mL for acute postoperative pain. The PCA device was preset to deliver a bolus dose of 0.5 mL with a lockout interval of 15 min and at a basal infusion rate of 2 mL/h. 

Adequate neuromuscular reversal was achieved by pyridostigmine 0.1–0.25 mg/kg and glycopyrrolate 0.05 mg per pyridostigmine 1 mg. Then, following extubation and relocation to the post-anesthesia care unit (PACU), the subjects received standard monitoring and care. Patients were moved to the general ward when they reached the discharge criteria with a modified postanesthetic Aldrete recovery score [14] of 10 which was assessed every 10 min. This recovery score consists of five parameters: oxygenation, respiration, circulation, consciousness, and motor activity, and each parameter is assigned a grade of 0–2. A modified postanesthetic Aldrete recovery score [14] of 10 implies SpO_2_ > 92% on room air, breathes deeply and coughs freely, blood pressure ± 20 mmHg of normal, fully awake, and moves all extremities. We did not record each score of parameters every 10 min in our case report form.

The intensity of postoperative nausea (PON) was rated on an 11-point verbal numerical rating scale (VNRS; 0 = without nausea, 10 = worst possible nausea). Based on the VNRS scores, PON severity was graded as mild (1–3), moderate (4–6), or severe (7–10). Postoperative vomiting was described depending on the appearance of vomiting or retching and recorded as present or absent. Postoperative pain was measured using the VNRS (0 = no pain, 10 = worst possible experienced pain). PONV and pain were assessed at three time points: at the PACU, 6 h after surgery, and 24 h after surgery. The highest VNRS score of pain and PON assessed every 10 min at PACU was used. The highest VNRS score of pain and PON from PACU discharge to 6 h after surgery mentioned by patients were recorded at 6 h after surgery and the highest VNRS score of pain and PON from 6 h to 24 h after surgery mentioned by patients were recorded at 24 h after surgery. Both the assessing anesthesiologist and patients were blinded to the group to which the participant was allocated.

As a rescue antiemetic, metoclopramide 10 mg was injected intravenously if the PON score was ≥4 or retching/vomiting occurred in the PACU or the general ward. If the PON score was ≥4 or retching/vomiting persisted or recurred within 6 h, IV chlorpheniramine 4 mg was added as a second-line rescue antiemetic. For rescue analgesia, IV fentanyl 50 μg once or twice (100 μg) was provided in the PACU if the pain score was ≥4 without severe PON or retching/vomiting. If the pain score was ≥4 with severe PON or retching/vomiting, ketorolac 30 mg or propacetamol 1 g was intravenously administered instead of fentanyl. Further, fentanyl was used if the severe PON or retching/vomiting subsided and the pain score persisted at ≥4. For pain control in the general ward, IV ketorolac 30 mg or propacetamol 1 g was administered, along with IV-PCA.

The primary outcome was the incidence of complete response, defined in this study as the absence of PONV without requiring rescue antiemetics until 24 h after operation. The secondary outcomes were the incidence and severity of nausea, incidence of retching or vomiting, and administration of rescue antiemetics.

Patient demographics, including the ASA Physical Status and detailed Apfel risk score for PONV (including female gender, non-smoker, history of PONV and/or motion sickness, and postoperative use of opioids, assigning 1 point for each presented factor) [2], were noted before the operation. Operative details, including the duration of anesthesia and emergence, were also noted. The surgeries included hysterectomy, myomectomy, ovarian cystectomy, oophorectomy, salpingectomy, and salpingo-oophorectomy.

### Statistical Analysis

As reported by our institutional medical records, the incidence of nausea in patients who were administered dexamethasone and ondansetron while undergoing laparoscopic gynecologic surgery with sufentanil-based IV-PCA was 50%. The required number of patients for a 50% decrement in the incidence of PONV [15,16] at 80% power, with a two-sided alpha error of 0.05, was 65 per group; thus, 144 patients were recruited, anticipating a dropout rate of 10%. The per-protocol approach was used to analyze the main results based on the data of the patients who completed this study. Statistical analyses were conducted with the SPSS Statistics software version 26.0 for Windows (IBM Corp, Armonk, NY, USA). For quantitative variables, normality was assessed using the Shapiro–Wilk test. The independent *t*-test or Wilcoxon test was used for the normally or non-normally distributed variables, respectively. For dichotomous variables, the χ^2^ test or Fisher’s exact test was used. Data are displayed as median (interquartile range [IQR]), or the number of patients (%). A *p*-value of less than 0.05 is considered to be statistically significant.

## 3. Results

**Figure 1:**
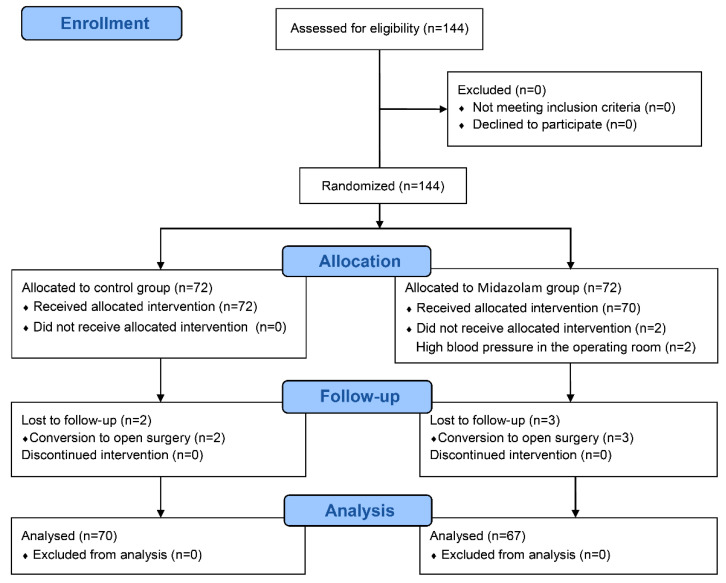
CONSORT flow diagram.

A total of 144 subjects were randomized after providing consent, and 137 subjects completed this trial (Figure 1). The patient characteristics including age, body mass index, ASA classification, and PONV risk factors were similar between the two groups. The operative details including the duration of emergence, and duration of PACU stay were also comparable. In the control group, the duration of anesthesia and the duration of surgery tended to be longer, which were not statistically significant (*p* = 0.06 for duration of anesthesia, *p* = 0.05 for duration of surgery) (Table 1). For the midazolam group, the mean injected midazolam dosage was 3.0 ± 0.5 mg, with no noted adverse effect.

The incidence of complete response during 24 h following laparoscopy was 41 (59%) in the control group and 48 (72%) in the midazolam group, and it was not significantly different between the two comparing groups (*p* = 0.11) (Table 2). The incidence of severe nausea at the three intervals was also similar between the two groups: transfer to the PACU, 2 (3%) vs. 2 (3%) (*p* = 1.00); from the PACU discharge to 6 h after surgery, 2 (3%) vs. 3 (5%) (*p* = 0.68); and from 6 h to 24 h after surgery, 4 (6%) vs. 1 (2%) (*p* = 0.37). Finally, the incidence of nausea, retching or vomiting, and the subjects who were administered rescue antiemetics at every time interval were comparable between the two groups.

The pain scores and subjects who were injected rescue analgesics at each time interval were similar between the two groups (Table 3). The dose of fentanyl injected in the PACU was also similar (26.4 ± 29.1 μg vs. 26.9 ± 30.6 μg, *p* = 0.93). One patient each from the control group and the midazolam group received 30 mg of ketorolac. One participant in the midazolam group received 1 g of propacetamol in addition to the previously administered ketorolac 30 mg in the PACU because of severe PONV. The pain diminished with these two non-opioid analgesics.

## 4. Discussion

This is the first randomized, double-blinded study to investigate whether midazolam would have an additive antiemetic effect on PONV when used with the dual prophylaxis of dexamethasone and ondansetron compared to the multimodal strategy of dexamethasone and ondansetron alone after gynecologic laparoscopy. In this trial, the addition of midazolam 0.05 mg/kg to dual prophylaxis was not superior to dual prophylaxis alone in terms of preventing PONV. The incidence of complete response 24 h after surgery, the incidence of nausea, severe nausea, retching/vomiting, and administration of rescue antiemetics were similar between the two groups.

The mechanisms of PONV include stimulation of the cortical/thalamic emetic center, vestibular nerve, and the chemoreceptor trigger zone, which lies on the floor of the fourth ventricle, exterior to the blood-brain barrier. Vagal stimulation of the gastrointestinal area is also a known mechanism. PONV is triggered by stimulating receptors such as 5-HT3 (serotonin), histaminergic (H1), muscarinic (M1), dopaminergic (D2), and neurokinin NK1 (substance P) and prevented or treated by targeting these receptors with antagonizing emetogenic substances [17,18]. Therefore, multimodal antiemetics with different receptor mechanisms are more effective than any single agent in preventing PONV [17,19]. Dexamethasone strengthens other antiemetics [8] by inhibiting the 5-HT3 expression in the neural tissue and its release in the gastrointestinal tract [20,21], inhibiting inflammatory mediators such as prostaglandins or substance P, and activating α2-adrenoreceptors [22]. Ondansetron is a highly selective 5-HT3 receptor antagonist, which works in the gastrointestinal tract as well as the chemoreceptor trigger zone, preventing serotonin action and inhibiting emesis [23]. The proposed mechanism of midazolam is the glycine mimetic inhibitory effect, which decreases 5-HT3 release by binding with the gamma-aminobutyric acid–benzodiazepine receptor complex, enhancement of the adenosinergic effect, suppression of dopamine release, and enhancement of the adenosine-mediated suppression of dopamine in the chemoreceptor trigger zone [24,25].

Several trials have reported the antiemetic characteristic of midazolam as a single agent. According to Bauer et al. [26], premedication using midazolam 0.04 mg/kg decreased the PONV frequency compared to placebo in outpatient surgery. Lee et al. [27] reported that the administration of 2 mg midazolam 30 min before the end of the operation had an antiemetic effect comparable to that of 4 mg ondansetron after minor gynecological and urological surgeries. Another study reported that administering midazolam 2 mg towards the end of surgery had an antiemetic effect similar to that of dual prophylaxis with IV dexamethasone 8 mg and IV ondansetron 4 mg. The PONV incidence within 24 h was 30% for the midazolam group and 33% for the dexamethasone and ondansetron group in patients undergoing laparoscopy, with an Apfel score ≥2, which was similar to the findings of our study [28]. Midazolam was also effective when used as a combination therapy with other classes of antiemetics. Midazolam 0.75 mg/kg coupled with ondansetron 4 mg before anesthetic induction reduced PONV effectively than ondansetron alone [12]. The combination of midazolam and dexamethasone before anesthetic induction has also been revealed to be more efficient than dexamethasone alone after middle-ear surgery for female patients [13]. Therefore, the antiemetic effect of midazolam may result from the activation of different receptors when compared with other antiemetics such as ondansetron or dexamethasone. As in the aforementioned studies, the frequently used midazolam doses for PONV were 2 mg or 0.04–0.075 mg/kg [12,13,26,27,28]. Grant et al. [10] showed similar effects between lower (<0.05 mg/kg) and higher doses (≥0.075 mg/kg) for preventing PONV, and we decided to use midazolam 0.05 mg/kg. The lower dose may not have been sufficient to elucidate the efficacy of midazolam as a third-line prophylaxis. The concerns for midazolam include postoperative sedation, cognitive delay, respiratory depression, and prolonged recovery time which are associated commonly with higher doses. Some previous studies have indicated that midazolam increased the PACU discharge time [29] and was linked to greater rates of respiratory depression in the PACU [30]. However, in this study, administering midazolam before anesthetic induction was not related to respiratory depression and delayed recovery time. In recent meta-analyses [10,11], the recovery time in the PACU was not affected by midazolam, which was consistent with our results. Other studies have shown that the emergence time was not prolonged by midazolam [31,32].

Since midazolam may act on different receptors compared to other antiemetics [10,33,34], we hypothesized that the addition of midazolam 0.05 mg/kg as pre-induction would be more efficient than dual prophylaxis of dexamethasone 4 mg and ondansetron 4 mg. Recent meta-analyses [10,11] revealed that midazolam was administered during pre-induction in many studies, and PONV incidence was not different significantly depending on the time of midazolam administration. Considering the results of earlier studies [12,13] and delayed recovery due to midazolam [29,30], we decided on the timing of midazolam administration before anesthesia induction.

Unlike previous studies, we used midazolam as a third-antiemetic strategy. Our study elucidated that the complete response of the control group (59%) was comparable to that of the midazolam group (72%) (*p* = 0.11). One possible explanation for this is that further reduction of PONV with triple prophylaxis (addition of midazolam) is more difficult than that with dual prophylaxis. Furthermore, ondansetron 12 mg was mixed with IV-PCA and administered continuously for preventing PONV in addition to administration of ondansetron 4 mg at the conclusion of surgery, which was performed often in South Korea [16,35] including our institution. This continuous postoperative therapy might present a strong bias when evaluating the preventive effect of midazolam on PONV. Apfel et al. [19] published an interesting factorial trial with 5161 patients, investigating the antiemetic efficacy of feasible compounds of two or three interventions using three antiemetics and three anesthetic methods. This study revealed that ondansetron, dexamethasone, and droperidol were equally effective in reducing the relative risk. However, the most important finding was that the effect of additional interventions was lesser than that of initial interventions regarding absolute risk reduction. Therefore, in our study, the effect of midazolam in patients who had already undergone two antiemetic interventions was too small to be considered as significant absolute risk reduction. 

Sugammadex may reduce PONV than pyridostigmine, which has muscarinic side effects that cause PONV, but this is still controversial [36,37]. Relatively high doses of pyridostigmine compared to anticholinergics can also cause PONV [36]. For reversal of neuromuscular blocker, pyridostigmine and glycopyrrolate were administered for all patients at a constant ratio, so the effect on PONV according to the reversal agent was not significant in this study.

The pain scores of the two groups were similar, 3.0 [2.0–4.0] for the control group and 4.0 [2.0–4.0] for the midazolam group, and 50% of the patients in both groups were administered rescue analgesics in the PACU because these patients had a pain score of ≥4, which was the standard for analgesic administration. This 50% is generally high despite implemented IV-PCA. However, this is similar to the previous study—47% of patients with IV-PCA administered additional analgesics in the PACU after laparoscopic hysterectomy [38].

This study has some limitations. First, as a result of the moderately inadequate sample size, we did not discover statistically significant distinctions in antiemetic prophylaxis between the control and midazolam groups First, in this study, the complete response of the midazolam group was 72%, which was similar to our goal of a complete response rate (75%), but nausea incidence (41%) of the control group in this study was lower than those (50%) of previous studies [15,16], which were used for sample size calculation. Due to this low incidence of nausea and relatively small sample size, this study could not achieve statistical significance in the difference in the anti-emetic effect between the control and midazolam groups. A further large-scale study is needed to facilitate generalization of our results. A previous retrospective study with 4057 patients found that midazolam was related to a decreased requirement for rescue PONV medication (midazolam group 12% [95% confidence interval = 11–13%] vs. no-midazolam group 15% [12–17%], *p* = 0.03) [9]. In this study, ondansetron and dexamethasone were administered as standard prevention in all patients, similar to our study. Moreover, a randomized multicenter trial of 1350 patients undergoing bowel surgery elucidated additional dexamethasone with standard PONV prophylaxis reduced PON 24 h after surgery (50% to 40%, *p* < 0.001) [39]. Second, PONV stratification was not precisely performed. Since our study involved female patients and opioid-based IV-PCA, the risk factors were two. However, as the risk factors increase, more combinations of antiemetics are recommended [4]. Therefore, strict risk stratification is recommended to evaluate the effect of midazolam. Moreover, sufentanil of IV-PCA was injected continuously at 0.05 μg/kg/h, and the exact time of bolus by the patient was not measured for a specific time interval for we used an elastomeric IV-PCA device. It would be more helpful to clarify the effect of postoperative opioid use on PONV by exact measurement of bolus times. Third, the duration of anesthesia and duration of surgery tended to be longer in the control group, which did not reach statistical significance. All patients underwent gynecologic laparoscopy after randomization, and the type of surgery was not different between the two groups. Therefore, it is questionable why this tendency of difference occurred. Duration of anesthesia especially using volatile anesthetics is a known risk factor for PONV [4,40], and although it is not statistically significant, the tendency of the difference in duration of anesthesia may have affected the PONV incidence.

## 5. Conclusions

The addition of midazolam 0.05 mg/kg at pre-induction to dual prophylaxis of dexamethasone and ondansetron had no additive preventive effect on PONV after gynecologic laparoscopy.

## Figures and Tables

**Table 1:** Patient demographics and operative details.

	Control Group (*n* = 70)	Midazolam Group (*n* = 67)	*p-*Value
Age (years)	39.0 [31.0–44.0]	37.0 [30.0–44.0]	0.93
ASA physical status (I/II)	58/12	51/16	0.44
BMI (kg/m^2^)	22.6 [20.8–25.6]	23.2 [20.9–25.8]	0.52
Non-smokers	57 (81%)	60 (90%)	0.27
Previous PONV or motion sickness history	31 (44%)	21 (31%)	0.17
Apfel’s risk score ^1^ for PONV			0.76
2	7 (10%)	7 (10%)	
3	38 (54%)	40 (60%)	
4	25 (36%)	20 (30%)	
Type of surgery			0.18
Hysterectomy	15 (21%)	15 (22%)	
Myomectomy	23 (33%)	13 (19%)	
Adnexal surgery ^2^	32 (46%)	39 (58%)	
Duration of anesthesia (min)	125.0 [100.0–155.0]	110.0 [92.5–130.0]	0.06
Duration of surgery (min)	87.5 [65.0–125.0]	75.0 [57.5–97.5]	0.05
Duration of emergence (min)	7.0 [5.0–9.0]	8.0 [6.0–10.0]	0.24
Duration of PACU stay (min)	55.0 [45.0–60.0]	55.0 [45.0–63.0]	0.43

Control group = dexamethasone and ondansetron were administered; midazolam group = midazolam, dexamethasone and ondansetron were administered. ^1^ Consists of female gender, non-smoker, history of PONV and/or motion sickness, and postoperative use of opioid. ^2^ Include ovarian cystectomy, oophorectomy, salpingectomy, and salpingo-oophorectomy. Values are presented as median [interquartile range], or number of patients (%). ASA, American Society of Anesthesiologists; BMI, body mass index; PONV, postoperative nausea and vomiting; PACU, post-anesthesia care unit.

**Table 2:** Postoperative nausea and vomiting.

	Control Group (*n* = 70)	Midazolam Group (*n* = 67)	*p*-Value
Nausea			
PACU	14 (20%)	5 (7%)	0.06
PACU discharge to 6 h after surgery	21 (30%)	12 (18%)	0.15
6 to 24 h after surgery	22 (31%)	13 (19%)	0.16
Severity of nausea (mild/moderate/severe)			
PACU	9/3/2	2/1/2	0.46
PACU discharge to 6 h after surgery	17/2/2	9/0/3	0.30
6 to 24 h after surgery	18/0/4	12/0/1	0.72
Retching or vomiting			
PACU	2 (3%)	2 (3%)	1.00
PACU discharge to 6 h after surgery	2 (3%)	2 (3%)	1.00
6 to 24 h after surgery	4 (6%)	1 (2%)	0.37
Rescue antiemetics			
PACU	5 (7%)	3 (5%)	0.72
PACU discharge to 6 h after surgery	4 (6%)	3 (5%)	1.00
6 to 24 h after surgery	4 (6%)	1 (2%)	0.37
PCA discontinuation	4 (6%)	1 (2%)	0.37
Complete response ^1^	41 (59%)	48 (72%)	0.11

Values are presented as number of patients (%). Control group = dexamethasone and ondansetron were administered; midazolam group = midazolam, dexamethasone and ondansetron were administered. ^1^ The definition is the absence of PONV without requiring rescue antiemetics until 24 h after surgery. PACU, post-anesthesia care unit; PCA, patient-controlled analgesia.

**Table 3:** Postoperative pain.

	Control Group (*n* = 70)	Midazolam Group (*n* = 67)	*p*-Value
Pain VNRS			
PACU	3.0 [2.0–4.0]	4.0 [2.0–4.0]	0.41
PACU discharge to 6 h after surgery	2.0 [2.0–3.0]	2.0 [2.0–3.0]	0.30
6 to 24 h after surgery	2.0 [1.0–2.0]	1.0 [1.0–2.0]	0.18
Rescue analgesics			
PACU	35 (50%)	33 (50%)	1.00
PACU discharge to 6 h after surgery	3 (4%)	3 (5%)	1.00
6 to 24 h after surgery	4 (6%)	7 (10%)	0.48

Values are presented as median [interquartile range] or the number of patients (%). Control group = dexamethasone and ondansetron were administered; midazolam group = midazolam, dexamethasone and ondansetron were administered. PACU, post-anesthesia care unit; VNRS, verbal numerical rating scale (0–10; 0 = no pain, 10 = worst possible experienced pain).

## Data Availability

The data presented in this study are available on request from the corresponding author.

## Abbreviations

5-HT3	5-hydroxytryptamine
ASA	American Society of Anesthesiologists
IQR	Interquartile range
IV	Intravenous
PACU	Post-anesthesia care unit
PCA	Patient-controlled analgesia
PON	Postoperative nausea
PONV	Postoperative nausea and vomiting
VNRS	Verbal numerical rating scale
